# The brain-liver cholinergic anti-inflammatory pathway and viral infections

**DOI:** 10.1186/s42234-023-00132-3

**Published:** 2023-12-20

**Authors:** Samuel Martínez-Meza, Bhavya Singh, Douglas F. Nixon, Nicholas Dopkins, Louie Mar A. Gangcuangco

**Affiliations:** 1 Division of Infectious Diseases, Department of Medicine, Weill Cornell Medicine, New York, NY USA; 2 Hawaii Center for AIDS, Department of Medicine, John A. Burns School of Medicine, University of Hawaii at Manoa, Honolulu, HI USA

**Keywords:** Vagus nerve, Viral infection, Liver-brain axis, Cholinergic signaling, Bioelectronic medicine

## Abstract

Efferent cholinergic signaling is a critical and targetable source of immunoregulation. The vagus nerve (VN) is the primary source of cholinergic signaling in the body, and partially innervates hepatic functionality through the liver-brain axis. Virus-induced disruption of cholinergic signaling may promote pathogenesis in hepatotropic and neurotropic viruses. Therefore, restoring VN functionality could be a novel therapeutic strategy to alleviate pathogenic inflammation in hepatotropic and neurotropic viral infections alike. In this minireview, we discuss the physiological importance of cholinergic signaling in maintaining liver-brain axis homeostasis. Next, we explore mechanisms by which the VN is perturbed by viral infections, and how non-invasive restoration of cholinergic signaling pathways with bioelectronic medicine (BEM) might ameliorate hepatic inflammation and neuroinflammation in certain viral infections.

## Introduction

The nervous system is a complex network of neurons and glial cells that permit rapid communication throughout the body by the release of soluble mediators. The nervous system is divided into two anatomically distinct components: the central nervous system (CNS), which includes the brain and the spinal cord, and the peripheral nervous system (PNS), which comprises all other nervous tissues (Waxenbaum et al. 2023). The PNS can be functionally distinguished into the autonomic nervous system (ANS), which controls involuntary physiological processes, and the somatic nervous system, which interprets sensory information and coordinates voluntary muscle movements (Waxenbaum et al. 2023). The ANS is composed of three anatomically distinguishable sub-systems: the sympathetic nervous system, the parasympathetic nervous system, and the enteric nervous system. There are two types of nerve fibers in the ANS: pre-ganglionic nerve fibers, which connect a central neuron with a ganglionic neuron, and post-ganglionic nerve fibers, which connect a ganglionic neuron to organs of the periphery. The ANS can be broken down into two phenotypically distinct neurotransmitter-based signaling systems, the cholinergic and adrenergic systems. In the cholinergic system, the post-synaptic release of acetylcholine (ACh) follows an action potential. In the adrenergic system, the post-synaptic release of catecholamines follows an action potential (McCorry 2007). In regarding to cholinergic signaling, pre-ganglionic fibers of both the sympathetic and parasympathetic nervous system utilize nicotinic ACh receptors (nAChRs). By contrast, post-ganglionic fibers in the parasympathetic division utilize muscarinic AChRs (mAChRs), whereas post-ganglionic fibers in the sympathetic division generally utilize adrenergic receptors (Waxenbaum et al. 2023).

A wide variety of cell types beyond those of a typical cholinergic synapse possess AChRs and other cholinergic system components (Fujii et al. 2017). Peripheral monocytes and lymphocytes both express AChRs that allow them to respond to efferent ANS signals (Fujii et al. 2017; Cox et al. 2020). ACh release by the VN can stimulate leukocytes which typically promotes an anti-inflammatory phenotype by upregulating the expression of immunoregulatory mediators (Tracey 2002). This change in immune cell functionality dependent on ACh release by the VN is termed the cholinergic anti-inflammatory pathway (CAP) (Tracey 2002; Pavlov et al. 2003). While inflammation underlies the bodily need to clear harmful stimuli, infectious agents, and cellular debris (Borovikova et al. 2000; Bernik et al. 2002), unregulated inflammation poses as a double-edged sword that further disrupts homeostasis to further drive disease-dependent pathophysiology (Santambrogio and Marrack 2023). Therefore, there are clinical needs for anti-inflammatory therapies to efficaciously combat pathological inflammation, regardless of source. Cholinergic signaling is one such understudied anti-inflammatory pathway that is targetable by non-invasive maneuvers (Borovikova et al. 2000; Bernik et al. 2002), making it an attractive alternative approach for the treatment of inflammatory disorders (Pavlov et al. 2003).

In organismal physiology, the CAP constitutes an immunoregulatory system designed to restrain pathological inflammation via VN-mediated activation of α7nAChR (Czura et al. 2007). CNS sensing of peripheral inflammation occurs through various mechanisms, such as vagal stimulation from cytokines released by VN-associated immune cells transmitted to the nucleus tractus solitarius (NTS). The NTS and its connections with the rostral ventrolateral medulla, area postrema, locus coeruleus, and the dorsal motor nucleus of the VN serve as key processors of peripheral inflammatory signals. In response to inflammation, the dorsal motor nucleus of the VN transmits efferent signals through the dorsal subdiaphragmatic VN to the celiac superior mesenteric ganglion complex, where synaptic transmission from the post-ganglionic sympathetic neuron reaches through the splenic nerve (Kressel et al. 2020). Stimulation of the splenic nerve incites the release of norepinephrine to local choline acetyltransferase (ChAT) positive T-cells in the spleen. ChAT + T Cells then release ACh, which acts on α7 nicotinic acetylcholine receptor (α7nAChR) positive splenic macrophages to alleviate inflammation (Pavlov et al. 2003; Rosas-Ballina et al. 2011; Rosas-Ballina et al. 2008). The VN extends its innervation not only to the spleen but also to most visceral organs. While VN mediated cholinergic signaling may directly regulate inflammation in many of these organs, it is also possible that the CAP may indirectly influence visceral organ immunity by exerting influence on the spleen, and therefore dictating systemic immune dynamics. Within this framework, the modulation of hepatic inflammation remains an appealing objective (Falvey et al. 2022). Currently however, it is not well understood how cholinergic signaling regulates hepatic inflammation.

BEM-based strategies are a promising treatment option to alleviate pathological inflammation (Tracey 2002). However, our understanding of the impact of CAP on certain pathologies, such as hepatic inflammation and viral infections, remains in infancy. In this mini-review, we outline the liver-brain axis is understudied in neurotropic and hepatotropic viral infections, and how restoration of the CAP may provide relief in the face of these infections.

## The liver-brain axis

Hepatic physiology controls host metabolism which in turn impacts behavior, while efferent signals from the CNS modulate metabolic function in the liver (Matsubara et al. 2022). This liver-brain axis relies upon signals propagated by the release of cytokines, chemokines, hormones, and neurotransmitters (Matsubara et al. 2022). Afferent innervation of this axis occurs through the NTS, as the VN stems from the medulla oblongata of the brainstem before exiting the skull through the jugular foramen, where it then exerts efferent parasympathetic functions throughout the host viscera and mediates motor, sensory, and metabolic functions (Kenny and Neuroanatomy 2023). Efferent innervation is transmitted through the hepatic branch of the VN to regulate hepatic metabolic function (McCracken et al. 2022; Waxenbaum et al. 2022; Metz and Pavlov 2018). Hepatic cholesterol and bile acid metabolism influence key components of the central nervous system, such as blood–brain barrier permeability and synaptic functionality (Yeo et al. 2023). Pathological perturbation of the liver-brain axis can alter functionality of both tissue sites. Hepatic encephalopathy, the presence of comorbid psychoses in patients with advanced liver diseases, is associated with pathological inflammation, microglial activation, and metabolic imbalances (Butterworth 2013). Similarly, nonalcoholic fatty liver disease (NAFLD) and non-alcoholic steatohepatitis (NASH) promote degenerative neuroinflammation (Kelty et al. 2023).

## The cholinergic system and hepatic immunity

Cholinergic signaling can be immunoregulatory in hepatic inflammation. Murine models demonstrate how α7nAChR expressing Kupffer cells respond to ACh secretion to alleviate immunopathologies in fulminant hepatitis (Li et al. 2014; Nishio et al. 2017).

In murine models of NASH, hepato-vagotomy worsens hepatic inflammation by upregulating tumor necrosis factor alpha (TNFα), interleukin 12 (IL-12), and monocyte chemoattractant protein 1 (MCP-1). Agonism of Kupffer cell α7nAChR alleviates symptoms of hepatic inflammation in NASH by suppressing cytokine production in a nuclear factor kappa-light-chain-enhancer of activated B cells (NFκB)-dependent manner (Nishio et al. 2017).

Concanavalin A (ConA) and lipopolysaccharide (LPS)-induced models of murine hepatitis further demonstrate the important of α7nAChR-mediated immunoregulation in Kupffer cells (Jo et al. 2020). In LPS driven hepatitis, left cervical vagotomy promotes higher levels of serum and liver-specific TNFα and IL-6 (Li et al. 2014). In ConA-induced liver injury, hepatic biomarkers of cellular damage such as alanine aminotransferase and aspartate aminotransferase are elevated upon disease induction, and subsequently ameliorated following bioelectronic stimulation of afferent VN fibers (Jo et al. 2020).

The activation of α7nAChR in Kupffer cells by vagal nerve stimulation (VNS) is shown to be partially dependent on the induction of the Src family of tyrosine kinases, which inhibit myeloid differentiation factor 88 (MYD88) (Li et al. 2014). Additionally, ACh-induced immune suppression of Kupffer cells is at least partially dependent on direct NFκB inhibition (Nishio et al. 2017), a pathway typically activated downstream of MYD88 signaling (Kawai and Akira 2007). It is currently unknown if the CAP-mediated inhibition of MYD88 and NFκB activity is enacted via orthogonal or non-orthogonal mechanisms. Collectively, these suggest that CAP can reduce inflammation by acting upon master the regulators of inflammation MYD88 and NFκB.

Cholinergic signaling likely influences other immune influencing cell types of the liver, such as stellate cells (Lam et al. 2008). Cholinergic stimulation of hepatic stellate cells promotes the fibrosis mediators transforming growth factor beta 1 (TGFβ1) and bone morphogenetic protein 6 (BMP-6) (Lam et al. 2008). Interestingly, murine models of zymosan-induced peritonitis, vagotomy increases the activation and raw abundances of hepatic stellate cells, suggesting a role for the VN in maintaining hepatic stellate cells quiescence (Ahmed et al. 2023).

Cholinergic signaling in the liver-brain axis appears to largely be dependent on splenocyte activity (Huston et al. 2006). Briefly, VN signals from celiac ganglia propagate via adrenergic fibers of the splenic nerve (Rosas-Ballina et al. 2008). Norepinephrine, generated upon VN stimulation, binds to β2-adrenergic receptors on splenic ChAT + CD4 + T cells (Rosas-Ballina et al. 2011), which serve as the principal ACh source upon activation. This mechanism presents a pathway by which the VN exerts anti-inflammatory effects without direct innervation of the liver (Liu et al. 2021).

Cholinergic signaling across the liver-brain axis likely impacts systemic physiology. Hepatic sensory afferents which connect the liver-brain axis are critical in maintaining intestinal homeostasis by controlling T cell populations (Teratani et al. 2020). Perturbation of the vagal sensory afferents nerve of the liver reduce the abundance of colonic peripheral regulatory T cells (pTreg cells) secondary to decreased retinoic acid synthesis and aldehyde dehydrogenase expression by intestinal antigen-presenting cells (Teratani et al. 2020). Moreover, it has been shown that disruption of the left VN sensory afferents from the liver in murine colitis reduce the colonic pTreg pool, collectively demonstrating the crucial importance of the VN in controlling the number of pTregs to maintain intestinal homeostasis (Teratani et al. 2020). Further studies in mice demonstrate that hepatic VN innervation in the liver affects gut microbial composition, impacting inflammation in the gut and cortex (Zhang et al. 2021). Collectively, hepatic inflammation is a critical component of organismal health, and emerging evidence demonstrates the importance of cholinergic-dependent immunoregulation in maintaining hepatic homeostasis.

## Viral perturbation of the ANS

Autonomic dysfunctions are a cluster of medical conditions related to dysregulation of the ANS. The ANS governs involuntary bodily functions such as heart rate, respiration, and digestion, and therefore autonomic dysfunctions severely impact human health and disease alike. Virus-associated autonomic dysfunction is characterized by a wide spectrum of clinical features, such as orthostatic hypotension, urinary dysfunction, visceral disease, and subclinical autonomic neuropathy (Carod-Artal 2018). Viral infections may perturbate autonomic and VN functionality through virus-induced cytotoxicity at autonomic centers (Jammoul et al. 2023) and by binding to the nAChRs of the VN (Rangon and Niezgoda 2022). While the impacts of viral life cycles on immunity have been extensively studied and discussed elsewhere (Palmer 2022), it is of timely importance to consider how viral infections might affect CAP to influence human health.

Virus-induced neurotoxicity has been observed in various components of the CNS and VN. In murine models of acquired immunodeficiency syndrome (AIDS) induced with LP-BM5, affect the splenic norepinephrine reuptake and auto-oxidation (Kelley et al. 2003). HIV-1 infection has been also associated with the upregulation of α7-nAChRs, facilitating intracellular calcium overload and neuronal cell death in the striatum (Capó-Vélez et al. 2018). These findings imply that HIV infection could potentially disrupt key CAP components, such as the VN or α7-nAChRs. Clinically, a higher prevalence of autonomic dysfunction is commonly observed among individuals living with advanced HIV-1 immunodeficiency (Carod-Artal 2018). Further work is needed on the potential influence of autonomic dysfunction in people living with HIV-1 (PLWH).

VN neuropathy has been observed in patients with COVID-19. SARS-CoV-2, the causative agent of COVID-19, has been theorized to be neurotropic and target various cranial nerves, including the VN, where infection may spread through infected nerve endings, retrograde transport, and transsynaptic transmission (Chen et al. 2022). Impaired vagal activity has been implicated as one of the underlying mechanisms of post-acute sequelae of SARS-CoV-2 infection, also known as “long COVID” (Acanfora et al. 2022).

In patients with viral hepatitis or NASH, intrahepatic anatomical nerve fiber thickness among biopsy specimens revels a decrease in the amounts of total nerve fibers and sympathetic nerve fibers (Mizuno et al. 2021). This decrease in the amount of nerve fibers likely has detrimental effects on ANS functionality, which might furthermore disrupt cholinergic signaling response viral hepatitis.

Collectively, viral infections disrupt multiple facets of the CAP. In the liver, where irreversible immunopathologies observed during hepatitis can be ameliorated with early immunotherapeutic interventions, there is indication of the impact of cholinergic signaling having a potentially protective effect. Our current understanding suggests that the liver-brain axis is a key instigator of hepatic immunoregulation in a manner that is dependent on cholinergic signaling from the spleen.

## Therapeutic potential targeting CAP in viral infections

VNS has been a long-standing adjunctive therapy for drug-resistant epilepsy (Penry and Dean 1990) and depression (Rush et al. 2005). As a non-invasive therapy, VNS is an increasingly attractive alternative to canonical pharmaceutical interventions that are associated with costs, risks, and adverse side effects (Aniwattanapong et al. 2022). As our understanding of the fundamental biology underlying BEM expands, so do the applications (Pavlov and Tracey 2022). Notably, the anti-inflammatory effect of VNS appears promising as a treatment for inflammatory disorders characterized by excess inflammation and immune dysregulation (Pavlov and Tracey 2022). Inflammatory disorders have no existing cure strategies and instead rely heavily on personalized regimens of immunosuppressive drugs (Fugger et al. 2020). These therapies are often efficacious, but are associated with side effects and resistances that develop with long-term use, highlighting the need for alternative or complementary therapies (Fugger et al. 2020).

In the liver, untreated chronic hepatic inflammation can lead to fibrosis and hepatocellular carcinoma (Tanwar et al. 2020), emphasizing the need for effective interventions in its early stages. Polypharmacy is seen among individuals with NAFLD and chronic liver disease, impacting quality of life (Patel et al. 2017). This further highlights the importance of exploring non-pharmacological alternatives to address hepatic inflammation. VNS has been shown in pre-clinical studies to be an effective strategy for reducing hepatic inflammation in experimental models of hepatitis by suppressing pro-inflammatory cytokine cascades (Li et al. 2014; Nishio et al. 2017; Jo et al. 2020). Orthogonally, a clinical trial among COVID-19 patients shows that C-reactive protein and procalcitonin decrease following VNS (Tornero et al. 2022). However, extensive searches of PubMed, ClinicalTrials.gov, and Google Scholar reveal no clinical studies that utilize BEM/VNS to treat hepatitis.

Recent evidence has shown that the pharmacological α7nAChR agonist “PNU-282987” promotes interferon production to inhibit the replication of enterovirus-71 in neural cells, contributing to neural protection via the JAK-STAT2 signaling pathway (Song et al. 2018). A pre-clinical study of the α7nAChR positive allosteric modulator “PNU-120596” found that the drug protects against neurotoxic viral proteins such as HIV-1 Tat, and therefore suggests that α7nAChR may be a key therapeutic target in HIV-neurocognitive disorders (Zhao et al. 2021).

## Conclusions

**Fig. 1:**
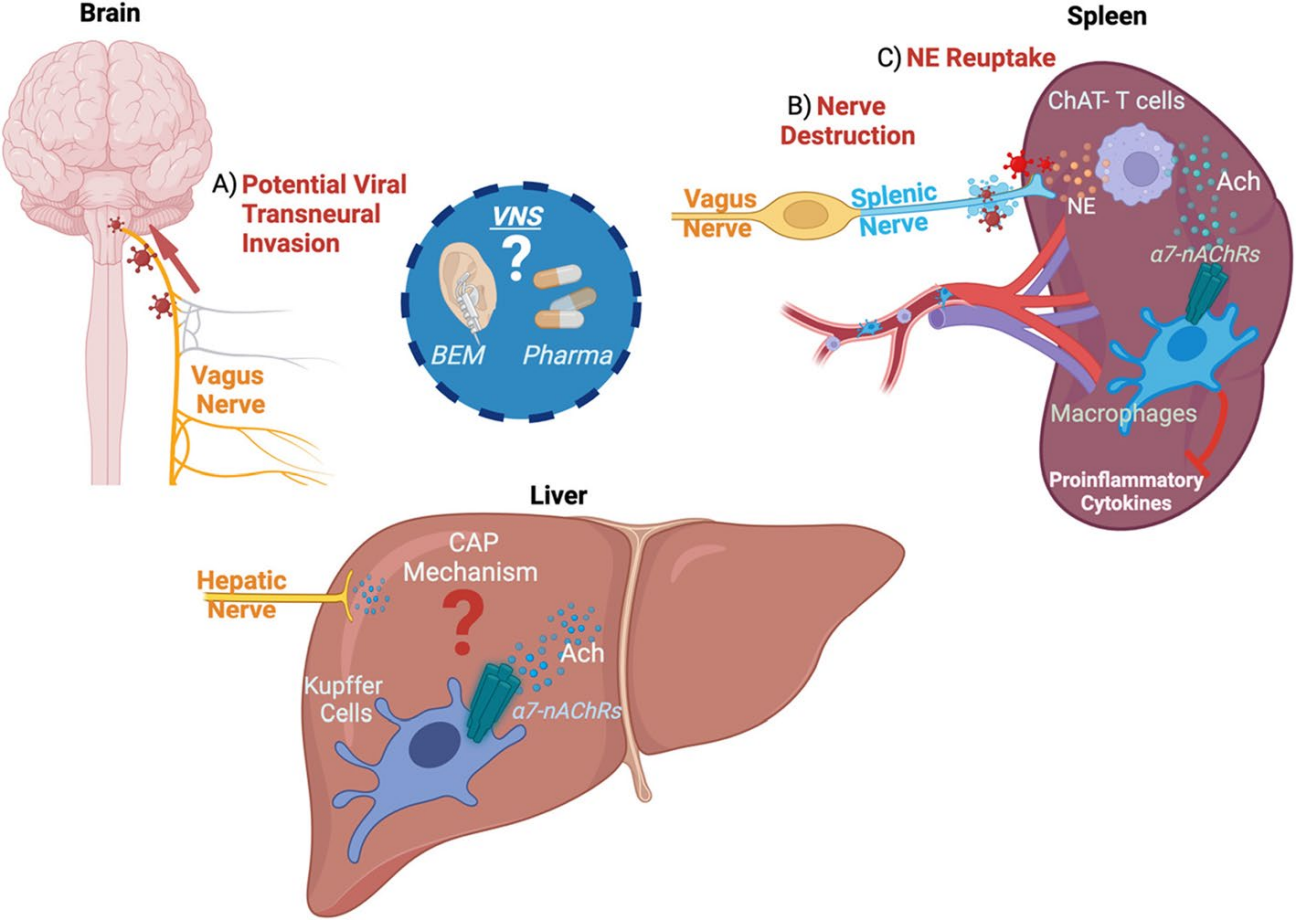
Proposed model of viral disruption to acetylcholine-mediated immune hepatic homeostasis. Essential components of the cholinergic anti-inflammatory pathway, including the molecular events in the spleen and liver are presented. Viruses such as SARS-CoV-2 potentially enter the central nervous system (CNS) through transneural migration via the vagus nerve (VN). Essential components of the cholinergic anti-inflammatory pathway, including the molecular events in the spleen and liver are presented (**A**). In HIV-1 infection, degradation of nerve fibers regulating norepinephrine (NE) uptake may contribute to autonomic and peripheral nervous system comorbidities commonly observed in people living with HIV-1 (PLWH) (**B** and **C**)

The VN plays a key role in the modulation of inflammation through the CAP. The COVID-19 and HIV-1 pandemics have spurred interest in the potential role of virus-induced autonomic dysfunction (Falvey et al. 2022; Rangon and Niezgoda 2022). Nevertheless, there is limited data on how viral infections affect the CAP and the liver-brain axis. Viruses may play a key role in CAP dysfunction, and therefore disruptions in cholinergic signaling might contribute to the inflammatory etiology of certain infections. Data on pharmacological interventions suggest potential benefits in targeting inflammatory pathways in the context of neurotropic and hepatotropic viral infections (Fig. 1). Targeted therapies through BEM are a rapidly developing therapeutic option for mitigating pathological inflammation, and further studies are warranted to investigate how VNS may restore immune homeostasis to improve clinical outcomes.

## Data Availability

N/A.

## Abbreviations

Ach: Acetylcholine
AChRs: Ach receptors
ANS: Autonomic nervous system
BEM: Bioelectronic medicine
CNS: Central nervous system
MCP-1: Chemoattractant protein 1
CAP: Cholinergic anti-inflammatory pathway
ChAT: Choline Acetyltransferase
ConA: Concanavalin A
HIV-1: Immunodeficiency virus
IL-: Interleukin
LPS: Lipopolysaccharide
NAFLD: Nonalcoholic fatty liver disease
MYD88: Myeloid differentiation primary response 88
NASH: Non-alcoholic steatohepatitis
PNS: Peripheral nervous system
pTreg cells: Peripheral regulatory T cells
TNFα: Tumor necrosis factor alpha
VN: Vagus nerve
VNS: Vagus Nerve stimulation
α7nAChR: α7 Nicotinic Acetylcholine Receptor
